# Telomere protein Rap1 is a charge resistant scaffolding protein in chromosomal bouquet formation

**DOI:** 10.1186/s12915-015-0149-x

**Published:** 2015-06-10

**Authors:** Hanna Amelina, Shaan Subramaniam, Vera Moiseeva, Christine Anne Armstrong, Siân Rosanna Pearson, Kazunori Tomita

**Affiliations:** Chromosome Maintenance Group, UCL Cancer Institute, University College London, London, WC1E 6DD UK

**Keywords:** Telomere, Shelterin complex, *Schizosaccharomyces pombe*, *Phosphorylation*, Meiosis

## Abstract

**Background:**

Chromosomes reorganize in early meiotic prophase to form the so-called telomere bouquet. In fission yeast, telomeres localize to the nuclear periphery via interaction of the telomeric protein Rap1 with the membrane protein Bqt4. During meiotic prophase, the meiotic proteins Bqt1-2 bind Rap1 and tether to the spindle pole body to form the bouquet. Although it is known that this polarized chromosomal arrangement plays a crucial role in meiotic progression, the molecular mechanisms of telomere bouquet regulation are poorly understood.

**Results:**

Here, we detected high levels of Rap1 phospho-modification throughout meiotic prophase, and identified a maximum of 35 phosphorylation sites. Concomitant phosphomimetic mutation of the modification sites suggests that Rap1 hyper-phosphorylation does not directly regulate telomere bouquet formation or dissociation. Despite the negative charge conferred by its highly phosphorylated state, Rap1 maintains interactions with its binding partners. Interestingly, mutations that change the charge of negatively charged residues within the Bqt1-2 binding site of Rap1 abolished the affinity to the Bqt1-2 complex, suggesting that the intrinsic negative charge of Rap1 is crucial for telomere bouquet formation.

**Conclusions:**

Whereas Rap1 hyper-phosphorylation observed in meiotic prophase does not have an apparent role in bouquet formation, the intrinsic negative charge of Rap1 is important for forming interactions with its binding partners. Thus, Rap1 is able to retain bouquet formation under heavily phosphorylated status.

**Electronic supplementary material:**

The online version of this article (doi:10.1186/s12915-015-0149-x) contains supplementary material, which is available to authorized users.

## Background

Telomeres are specialized nucleoprotein structures that form the natural ends of linear chromosomes. While telomeres are mostly known for their essential function in chromosome maintenance, they also play an important role in meiotic progression [1]. During meiotic prophase, the position of chromosomes within the nucleus is dramatically reorganized and telomeres cluster within a limited area of the nuclear envelope to form the so-called telomere bouquet [2, 3]. This conserved reorganization of chromosomes during meiotic prophase has been observed in most eukaryotic organisms and is shown to promote homolog pairing and meiotic recombination [4]. Recent studies suggest that the bouquet also plays a crucial role in meiotic spindle formation [5].

In fission yeast *Schizosaccharomyces pombe,* the telomere bouquet is observed throughout meiotic prophase. This period is also known as the ‘horsetail nucleus’ stage, during which the nucleus elongates and oscillates back and forth between the cell poles, following the spindle pole body or SPB (the yeast equivalent of the centrosome) driven by cytoplasmic microtubules [6]. Bouquet formation is achieved by expression of a pair of meiosis-specific proteins, Bqt1 and Bqt2, which bridge the telomeric proteins Taz1 and Rap1 to the SPB component Sad1 [7]. To ensure telomere attachment to the SPB, telomeres must be tethered to the nuclear envelope via the interaction between Rap1 and the inner nuclear membrane complex Bqt3 and Bqt4 [8]. Disruption of any of these components leads to failure of telomere clustering and defective spore formation in meiosis [7–11]. Sporulation defects in the bouquet mutants occur mainly due to impaired spindle formation and partly due to compromised meiotic centromere assembly, followed by chromosome segregation defects [5, 12]. Recent studies suggest that recruitment of a single telomere tract or centromere to the SPB is sufficient to confer functional spindles [13, 14]. Hence, the telomere bouquet does not only function in alignment of chromosomes but is also crucial for the recruitment of a chromosome to the SPB to create a functional meiotic spindle.

Although the bouquet composition and its function are becoming better understood, the molecular mechanisms that govern dissociation of telomeres from the SPB remain elusive. Interestingly, in fission yeast, polarized bouquet configuration is maintained until the end of meiotic prophase, and upon entry into the first meiotic division, telomeres dissociate from the SPB in a concerted manner, dubbed ‘telomere fireworks’ [5]. Another interesting observation is that Bqt1 and Bqt2 do not localize to telomeres at the onset of the first meiotic division [7]. Moreover, Rap1 is highly phosphorylated in mitotic cells [15]. We therefore hypothesized that disruption of the interaction between Rap1 and the Bqt1-2 complex, potentially by means of post-translational modifications of one or both interacting partners, may be responsible for telomere dissociation from the SPB. In this report, we investigated whether Rap1 is involved in the termination of the telomere bouquet. Comprehensive phosphoproteomic analysis of the meiotic Rap1 protein revealed that it is progressively phosphorylated throughout meiotic prophase and the number of phosphosites peaks after completion of the bouquet stage. Surprisingly, this massive phosphorylation of Rap1 is dispensable for telomere bouquet dissociation, as indicated by our live microscopy analysis and protein interaction studies of the phospho-mutants. Our study illuminates that Rap1 is a protein resistant to negative charge and functions as a ‘scaffolding’ protein in the telomere bouquet.

## Results

### Rap1 is hyper-phosphorylated in meiosis

**Fig. 1:**
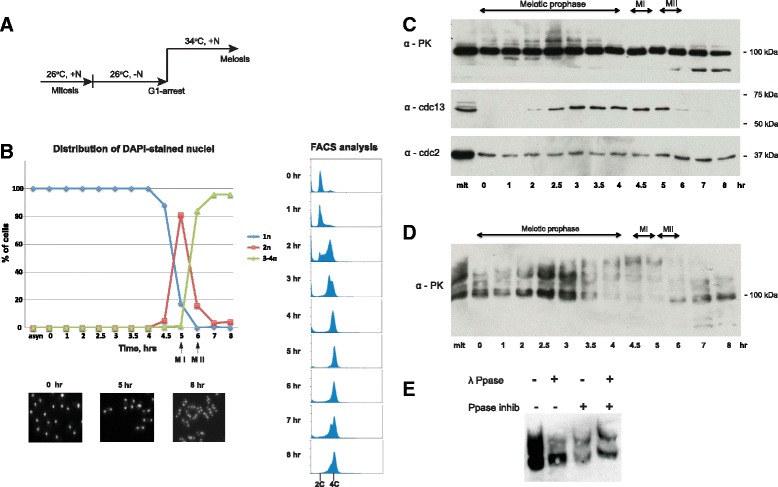
Rap1 is hyper-phosphorylated in meiosis. **a** Schematic diagram of meiotic culture synchronization using homozygous diploid cells carrying the temperature-sensitive *pat1-114* mutation and the *mat-Pc* cassette. **b** Distribution graph of the number of nuclei in meiocytes through meiosis (top left) and images of DAPI-stained cells from indicated fractions (bottom left). FACS analysis shows DNA duplication from 2C to 4C (right). **c**,**d** Western blot analysis of Rap1-3xPK from mitotic cycling cells (mit), G1 arrested cells (time 0) and meiotic cell fractions at indicated times. Anti-Cdc2 (CDK) and anti-Cdc13 (Cyclin B) antibodies were used as a loading control and meiosis synchronicity marker, respectively. **c** Separation of cell extracts on a standard gradient gel. **d** Separation of phosphorylated Rap1-3xPK on a Phos-tag gel. **e** Treatment of Rap1-3xPK from the 4.5 hr fraction with lambda-phosphatase and/or phosphatase inhibitors as a control. Note that fast-migrating bands of Rap1 are observed in this case due to the presence of endogenous phosphatases

To investigate the stability of the Rap1 protein throughout meiosis, a homozygous diploid (*h*^*−*^*/h*^*−*^) temperature-sensitive *pat1-114* strain carrying a *mat-Pc* cassette was utilized to synchronize meiosis [16, 17]. Meiosis was induced after nitrogen starvation, followed by a temperature shift from permissive (26°C) to restrictive conditions (34°C) (Fig. 1a). Progression of meiosis was monitored by assessing the number of nuclei and DNA content per cell from fractions collected at 30-minute or 1-hour intervals during the synchronization procedure (Fig. 1b). In order to assess Rap1 protein stability during meiosis, Rap1 was endogenously tagged with PK (V5) epitope peptide and detected by anti-V5 antibodies. Western blotting analysis of synchronous culture extracts showed that Rap1 protein is rather stably expressed during meiosis, although lower molecular weight, potentially truncated forms of Rap1, were observed at the end of meiosis (Fig. 1c, top panel).

Interestingly, a number of distinctly shifted bands of Rap1 were detected during meiotic prophase. Similar shifted bands of Rap1 have also been recently reported [18]. To determine if Rap1 is phosphorylated during meiosis, cell extracts were further analyzed using Phos-tag™ SDS-PAGE [19]. Phos-tag™ gel analysis revealed that the Rap1 protein is highly phosphorylated during meiosis. Strikingly, the maximum level of phosphorylation was observed at 4.5–5 hr, when almost none of the fast-migrating forms of Rap1 were detected (Fig. 1d). Phosphatase treatment confirmed that the shifted bands observed at 4.5–5 hr represented phosphorylated forms of Rap1 (Fig. 1e). Thus, our data indicates that Rap1 phosphorylation accumulates as meiotic prophase progresses, and Rap1 becomes hyper-phosphorylated at the onset of meiosis I, when the bouquet stage ends [5].

### Mass spectrometry analysis of Rap1 reveals an increasing number of phosphosites detected upon completion of the bouquet stage

**Fig. 2:**
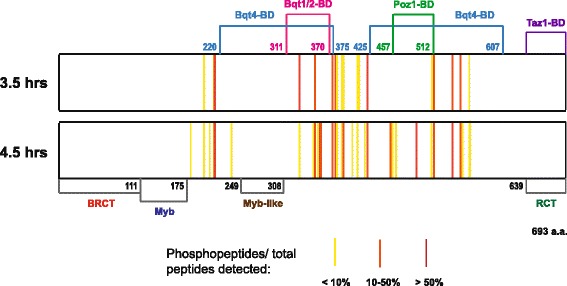
Domain organization and schematic of phosphorylation sites of Rap1 protein detected at 3.5 hr and 4.5 hr into meiosis. Phosphorylation sites are highlighted as bars with a colour code (yellow, less than 10%; orange, 10–50%; and red, over 50%). Protein interaction domains are indicated above and the structural domains are shown at the bottom. BRCT, BRCA1 C-terminus domain; Myb domain, Myb-like domain; RCT, Rap1 C-terminus domain

To determine the location of phosphorylation sites in Rap1, meiotic Rap1 was purified from fractions of the synchronized culture at 3.5 hr and 4.5 hr, and was subjected to mass spectrometry analysis. Using trypsin digestion, we covered 70–75% of the Rap1 protein sequence at 95% peptide threshold, and identified 19 and 35 phosphorylation sites from 3.5 hr and 4.5 hr, respectively (Additional file 1). Notably, all phosphorylated sites identified at 3.5 hr were also detected at 4.5 hr, suggesting that Rap1 phosphorylation accumulates with progression of meiotic prophase. Our analysis revealed several meiosis-specific phosphorylation sites in addition to those detected in mitosis-arrested cells [15]. With respect to known protein binding domains of Rap1 [15, 20], the phosphorylated sites at 3.5 hr (early prophase) fell into three clusters, whereas at 4.5 hr phosphosites were fairly evenly distributed across Rap1 (Fig. 2). Interestingly, two and six phosphosites were identified in the Bqt1-2 binding area (311–370 amino acids) at 3.5 hr and 4.5 hr, respectively. Notably, although phosphorylations were detected within the Bqt4 and Poz1 binding regions, we did not detect any phosphorylation within known structural domains of Rap1. Altogether, our mass spectrometry data suggest that the number of phosphorylated residues of Rap1 increases with the progression of meiosis, which is in agreement with our Phos-tag™ gel analysis (Fig. 1d).

### Hyper-phosphorylation of Rap1 in meiosis is dispensable for telomere bouquet formation and dissociation

**Fig. 3:**
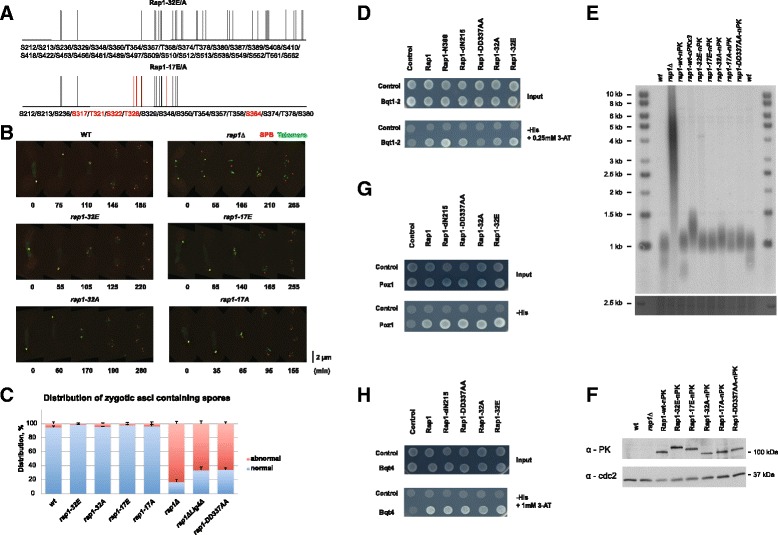
Rap1 hyper-phosphorylation in meiosis is dispensable for telomere bouquet clustering/dissociation. **a** Schematic of phosphomimetic and unphosphorylatable cluster mutants of Rap1 created and analyzed in this study. Additional mutation sites introduced in 17E/A (see text) are highlighted in red. **b** Series of frames from films of meiosis. The SPB and telomeres were observed via endogenously tagged Sid4-mCherry and Taz1-YFP, respectively. Time count starts from the beginning of filming. Scale bar equals 2 μm. Example of defective meiotic SPB is shown in *rap1*∆. None of the *rap1* cluster mutants exhibit defective SPB (examined cell number of indicated strains is more than 20). **c** Frequency of normal four-spore asci in *rap1* phosphomutants. Zygotic asci generated from the indicated genotypes in an *h* ^*90*^ (homothallic) background were scored by light microscopy. Two hundred asci per genotype were counted in each experiment. Data represent the average of three experiments. Error bars indicate standard deviations. **d**,**g**,**h** Yeast two-hybrid analysis of the interaction between mutant Rap1 and the (**d**) Bqt1-2 fusion protein, (**g**) Poz1 and (**h**) Bqt4. **e** Telomere lengths of the *rap1* phosphomutants. Telomere Southern blot of genomic DNA digested with *Eco*RI and hybridized with a telomeric probe. A fragment of the SafeView Nucleic Acid Stain stained gel image at 2.5 kb is shown below the blots as a loading control. **f** Protein expression levels of the N-terminal PK-tagged mutant Rap1. SPB, spindle pole body

Since we observed that Rap1 phosphorylation peaks at meiosis I, we speculated that the resulting highly negative charge of Rap1 is responsible for the change in its affinity to the Bqt1-2 complex. In order to mimic hyper-phosphorylated Rap1, all validated phosphorylation sites from S-212 to S-562 were substituted with negatively charged glutamate residues (*rap1-32E*) (Fig. 3a). To monitor telomeres and the SPB through meiosis, endogenous Taz1 and Sid4 were tagged with YFP and mCherry, respectively. To our surprise, the phosphomimetic *rap1-32E* mutants did not exhibit any detectable meiotic defects and their telomeres clustered and dissociated from the SPB in a timely manner very similar to that of the wild-type (Fig. 3b). Accordingly, *rap1-32E* mutants exhibited no sporulation defects (Fig. 3c). The corresponding non-phosphorylatable mutant form of Rap1 (*rap1-32A*) also did not cause defects in meiotic progression and telomere bouquet behaviour (Fig. 3a,b,c). Western blot analysis from meiotic cell extracts confirmed that the mutant forms of Rap1 were stably expressed, and the phospho-modification of Rap1-32A was significantly reduced (Additional file 2). Finally, our yeast two-hybrid assay confirmed that the Bqt1/2 binding domain of Rap1 falls within 216–388 amino acids, and introduced cluster mutations did not affect its interaction with the Bqt1-2 complex (Fig. 3d).

Suspecting that some phospho-modifications might remain unidentified in our study, five additional serine and threonine residues (S-317, T-321, S-322, T-328 and S-364), along with 12 detected phosphosites within and adjacent to the Bqt1/2 binding domain, were all substituted to glutamate (*rap1-17E*) or alanine (*rap1-17A*) (Fig. 3a). However, these mutations also did not cause any defects in meiosis (Fig. 3b,c). Thus, we conclude that accumulation of negative charge at the Bqt1-2 binding domain of Rap1 does not affect its ability to form the bouquet.

Because *rap1-32A* and *rap1-32E* bear mutations within the binding domain of the telomerase negative regulator Poz1 [21], we checked whether telomere length regulation was impaired in the *rap1* phospho-mutants. Since C-terminus tagging of Rap1 impaired telomere length homeostasis (Fig. 3e), the PK epitope tag was fused to the N-terminus. Although phosphomimetic forms of the Rap1 protein (Rap1-32E and 17E) migrate slower than wild-type Rap1, none of the cluster mutations affected protein stability (Fig. 3f). The strains carrying mutant Rap1 maintained their telomere length comparable to that of wild-type (Fig. 3e). Accordingly, all mutants retained their ability to interact with Poz1 by the yeast two-hybrid assay (Fig. 3g). Additionally, both *32A* and *32E* mutant forms of Rap1 retained the ability to interact with Bqt4 (Fig. 3h). Indeed, telomere localization to the nuclear periphery in interphase was not impaired in *rap1-32A* and *32E* mutants (Additional file 3). Thus, hyper-phosphorylation of Rap1 observed in meiosis does not appear to have a role in telomere bouquet regulation. Furthermore, our mutagenesis analysis suggests that Rap1 is able to resist high negative charge changes without affecting its function in meiosis or telomere length homeostasis.

### Intrinsic negative charge of Bqt1/2 binding domain of Rap1 is crucial for telomere bouquet formation

**Fig. 4:**
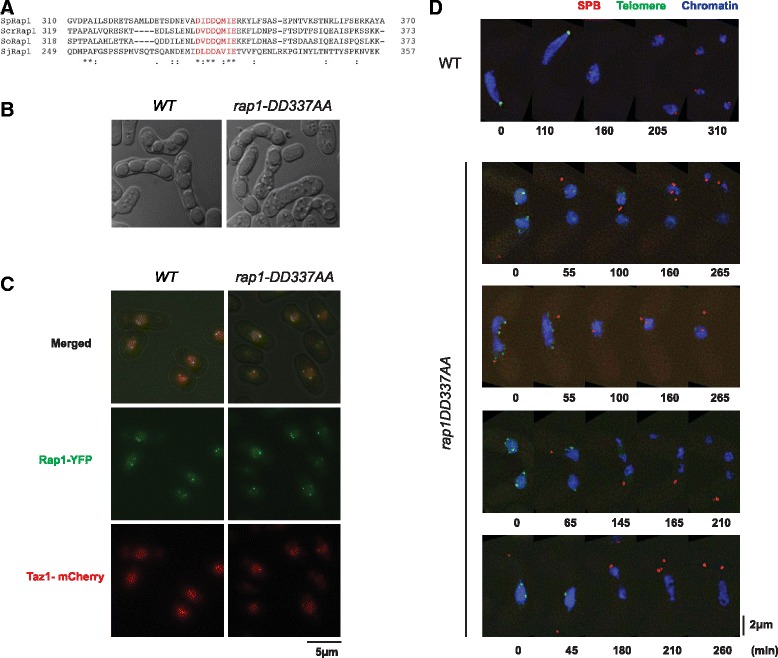
Negative charge of Rap1 Bqt1/2 binding domain is important for functional telomere bouquet. **a** Alignment of Rap1 protein sequences from different fission yeast species, highlighting a highly conserved area within Rap1 (performed using Clustal Omega; EMBL-EBI, Cambridge, UK). Sp, *Schizosaccharomyces pombe*; Scr, *S. cryophilus*; So, *S. octosporus*; Sj, *S. japonicus*. **b** Morphology of zygotic asci of *rap1-DD337AA* mutant compared to cells expressing wild-type Rap1. Frequency of normal four-spore asci is shown in Fig. 3b. **c** Mitotic cells expressing Rap1-YFP or Rap1(DD337AA)-YFP (green in the merged pictures) and Taz1-mCherry (red in the merged pictures). Both wild-type Rap1 and Rap1-DD337AA co-localize with Taz1-mCherry. Scale bar equals 5 μm. **d** Series of frames from films of meiosis. The SPB, telomeres and chromosomes were observed via endogenously tagged Sid4-mCherry, Rap1-YFP and Hht1-Cerulean, respectively. Time count starts from the beginning of filming. Scale bar equals 2 μm. Top image, wild-type cells; and bottom four images, examples of defective meiosis in *rap1-DD337AA*. Note that *rap1-DD337AA* meiosis is reminiscent of *rap1*∆ meiosis shown in Fig. 3b

Rap1 protein is negatively charged, and the Bqt1/2 binding region is particularly rich in hydrophobic and negatively charged amino acid residues. Some of these negatively charged residues (D-335, D-337, D-338 and E-342) are well-conserved among fission yeast species (Fig. 4a). Importantly, mutation analysis indicated that Rap1-DD337AA (*D337A* and *D338A* mutations) no longer interacts with the Bqt1-2 complex, but retains its ability to interact with Bqt4 and Poz1 in yeast two-hybrid assay (Fig. 3d,g,h).

To study the function of Rap1-DD337AA, endogenous *rap1* was mutated and fused to YFP. Accordingly, *rap1-DD337AA* mutants were defective in sporulation (Figs. 3c and 4b). Live cell imaging of the mutant showed that Rap1-DD337AA localized to telomeres, as determined by co-localization to Taz1 (Fig. 4c), but did not cluster at the SPB in meiotic prophase (Fig. 4d). Furthermore, in many cases the SPB was destabilized and detached from the nucleus and, as a consequence, aberrant chromosome segregation was observed (Fig. 4d). These meiotic phenotypes are characteristic of *rap1*∆ mutants as well as the bouquet-defective mutants [5]. However, Rap1-DD337AA was stably expressed and telomere length of the *rap1-DD337AA* mutant was the same as that of wild-type (Fig. 3e,f). Additionally, telomeres of the mutant cells were retained at the nuclear periphery in interphase (Additional file 3). Thus, *rap1-DD337AA* is a meiosis-specific loss-of-function mutation, and negatively charged aspartates at positions 337 and 338 are crucial for bouquet formation.

## Discussion

In this study we have shown that the level of phosphorylation of Rap1 gradually increases during the course of meiotic prophase, peaking in meiosis I. The SPB is known to recruit a number of kinases and phosphatases that modify its subunits, and these modifications play a critical role in regulating mitotic commitment and meiotic progression [22–25]. Since telomere heterochromatin comes into close contact with the meiotic SPB during chromosomal bouquet configuration, the telomere-bound proteins are likely to be exposed to these kinases. Additionally, DNA damage checkpoint kinases are activated during meiotic recombination [26]. In fact, some phosphorylations originate from telomere-associated kinases as indicated by reduced shifted bands of Rap1 at 4–4.5 hr in the absence of Taz1 (Additional file 4). Nevertheless, our study suggests that the hyper-phosphorylation of Rap1 observed during meiosis is not directly involved in the regulation of the bouquet. Rap1 can withstand significant charge changes that do not affect interactions with its binding partners and its function in meiosis. Hence, rather than a functional regulatory protein, meiotic Rap1 appears to be a ‘scaffolding’ protein, that is targeted by multiple kinases. Interestingly, while mutating 32 phosphorylation sites did not alter Rap1 function, mutation of only two highly conserved residues (D-337 and D-338) disrupted its ability to bind to the Bqt1-2 complex, causing pronounced defects in telomere clustering and chromosome segregation. In contrast, the binding partners Bqt1 and Bqt2 are positively charged, suggesting that Rap1 binds to the Bqt1-2 complex through hydrogen bonding interactions. Thus, we speculate that negatively charged residues at Rap1’s interaction surfaces are evolutionarily conserved in order to retain affinity under shifts in charge occurring throughout meiotic prophase.

Rap1 is also highly phosphorylated in the mitotic cell cycle, particularly in M-phase. Among the phosphorylation sites reported for mitotic Rap1, five phosphorylation sites (S-213, T-378, S-422, S-456 and S-513) were shown to have an inhibitory effect on Rap1-Bqt4 interaction, which was demonstrated by the phosphomimetic *rap1-5D/5E* mutants [15]. In mitosis, Cdc2 phosphorylates three of these sites in order to temporarily release telomeres from the nuclear envelope. This mechanism assists faithful chromosome segregation in anaphase. However, *rap1-5D* and *rap1-5E* mutants do not have any sporulation defects [15], which suggests that the bouquet is intact. This is surprising since the Rap1-Bqt4 interaction is required for telomere clustering in meiosis [8], and raises the possibility that telomeres remain associated with Bqt4 and the SPB via different mechanisms. Notably, four residues of Rap1 including Cdc2 targets were also found to be phosphorylated throughout meiotic prophase in our study (except for S-456, detected only at 4.5 hr) (Additional file 1). Unlike the Rap1-5E mutant protein, our phosphomimetic cluster mutant Rap1-32E*,* which includes 5E mutation (Fig. 3a), was able to interact with Bqt4. Thus, we predict that pre-meiotic hyper-phosphorylation of Rap1 (or particular phosphorylations among them) counteracts Cdc2 kinase action to preserve affinity to Bqt4, and thereby maintain telomere localization to the nuclear membrane and bouquet configuration.

## Conclusions

Rap1 hyper-phosphorylation observed during meiotic prophase does not have a direct role in telomere bouquet regulation. Rap1 uses its negatively charged amino acid residues to bind the Bqt1-2 complex. Therefore the interaction is not affected by changes in net charge caused through progressive hyper-phosphorylation.

## Methods

### Yeast genetics and plasmids

The genotypes of the strains used for this study are listed in Additional file 5. All media and supplements were purchased from Formedium™ (Hunstanton, UK). Fission yeast was grown at 32°C in standard rich media (YES) unless indicated. Epitope tag insertion at the C-terminus was described previously [27]. A plasmid for N-tagged *rap1* was constructed by cloning the *rap1* gene, including 1,360 bases of the upstream and 1,500 bases of the downstream regions, with a primer set including a *Kpn*I site. The start codon of *rap1*^*+*^ was replaced with a single V5 (PK) sequence. A *kanMX6* cassette was inserted 700 bases upstream of the gene. The resulting plasmid pRap1a-nPK was digested with *Kpn*I and replaced the *ura4*^+^ cassette at the *rap1* gene locus in *rap1*∆ cells. The transformants were backcrossed with a wild-type strain and cultured for 2 weeks before analysis of telomere length. To generate *rap1* mutants, cluster-mutated *rap1* gBlocks were synthesized (Integrated DNA Technologies, Coralville, IA, USA) and replaced wild-type *rap1*^+^ in the pRap1a-nPK plasmid and the yeast two-hybrid pGAD and pGBK plasmids.

### Yeast two-hybrid assays

The assay was conducted according to the Matchmaker Gold Yeast Two-Hybrid System manual (Clontech, Mountain View, CA, USA). Expression vectors for the GAL binding domain (BD) fused proteins and the GAL activation domain (AD) fused proteins were generated by subcloning of the indicated cDNAs into pGBK and pGAD, respectively. Expression of BD and AD fused proteins were confirmed by Western blotting using anti-myc and anti-HA antibodies, respectively. To express Bqt1 and Bqt2 together in the yeast two- hybrid plasmids, *bqt2*^+^ cDNA was inserted at the start codon of the *bqt1*^+^ cDNA sequence; the resulting plasmid was named pGBK-Bqt2-1.

### pat1-114 synchronization

Cells were first cultured in YE media at 26°C overnight until late stationery phase, then transferred to EMM media containing a nitrogen source and incubated for 24 hr until mid-log phase (OD = 0.5–0.7). The cells were next transferred to EMM media without a nitrogen source (EMM-N) by filtering, and cells were incubated for another 15–16 hr to arrest cells at G1 phase. To inactivate the *pat1* kinase gene and induce meiosis, the temperature was shifted up to 34°C, cultures were supplemented with one-fifth volume of EMM media pre-warmed to 34°C, and meiotic fractions were collected at the required time point.

### Western blotting and Phos-tag gel

Whole-cell protein extracts prepared using a trichloroacetic acid method were separated by SDS-PAGE using 10% acrylamide gels. Western blotting was performed with anti-V5 peptide (Bio-Rad, Hercules, CA, USA), anti-Cdc2 (Santa Cruz Biotechnology, Dallas, TX, USA) and anti-Cdc13 (Santa Cruz Biotechnology) antibodies following a standard protocol. For detection of phosphorylated Rap1 forms, 7.5% acrylamide gels were supplemented with 25 μM PhosTag ligand (AAL-107; NARD Institute, Amagasaki, Japan) and 50 μM MnCl_2_, according to the protocol [19]. A Phos-tag gel was treated with 1 mM EDTA prior to transfer. Two technical replicates of the Western blotting image in Fig. 1c are shown in Additional file 6.

### Telomere Southern blotting

Southern blotting was performed as described previously [27]. Equal amounts of *Eco*RI-digested DNA fragments were separated on a 1% agarose gel and subjected to Southern blotting with a telomere probe.

### Preparation of cell extracts and immunoprecipitation for mass spectrometry

Six litres of synchronous meiotic cells expressing Rap1-3xPK were harvested at 3.5 hr (early prophase) or 4.5 hr (late prophase) following the *pat1-114* synchronization protocol as described above. Rap1-3xPK was immunoprecipitated from native cell extracts in RIPA buffer (50 mM Tris–HCl, pH 8; 150 mM NaCl; 1% NP-40; 0.5% sodium deoxycholate, and 0.1% SDS), supplemented with 1X PhosSTOP (Roche, Basel, Switzerland); 1× cOmplete EDTA-free protease inhibitor (Roche); 1 mM PMSF, 1 mM DTT, 0.1 ng/mL MG132 (Sigma-Aldrich, St Louis, MO); and 10 U/mL TURBO DNase (Ambion, Life Technologies, Carlsbad, CA, USA), using Dynabeads M-270 epoxy (Life Technologies) pre-coated with anti-V5 peptide antibodies.

### Phosphatase treatment of native cell extracts

Native whole-cell extracts were prepared using modified HB buffer (50 mM HEPES-KOH, pH 7.5; 140 mM NaCl, 0.1% NP-40; 1 mM MnCl_2_). Then 20 μl of lysate was treated with 4 μl of lambda-phosphatase; 10 μl of phosphatase inhibitors; both phosphatase and inhibitors; or water as a control. The samples were incubated at 30°C for 1 hour before being separated on SDS-PAGE gel.

Further details about microscopy and mass spectrometry methods can be found in Additional file 7. A list of phosphopeptides detected by mass spectrometry is shown in Additional file 8.

### Open access of data

The mass spectrometry proteomics data have been deposited to the ProteomeXchange consortium [1] via the PRIDE partner repository with the dataset identifier PXD001841.

## Additional files

Additional file 1:
**List of Rap1 phosphorylation sites identified in the study.**
Additional file 2:
**Western blot images of TCA-extracted Rap1 proteins from meiotic cells. Indicated cells were induced to undergo meiosis using the** ***pat1-114*** **mutation, and were collected from the 4.5 hr fraction.** Cell extracts were separated in regular gradient (left) and Phos-tag SDS-PAGE (right), and subjected to Western blot using anti-PK(V5) antibody. Unlike wild-type meiotic Rap1 that shows multiple bands, Rap1-32A exhibits a single band in the Phos-tag gel, suggesting the phospho-modifications were diminished in this mutant form. Additional file 3:
**Localization of telomeres in mitotic interphase.** (**A**) Projected images of cells in mitotic interphase. Taz1-YFP, Sid4-mCherry and Bqt3-Cerulean were used to visualize telomeres, spindle pole body and the membrane, respectively. White and grey arrows indicate examples of telomeres localized at the nuclear periphery and away from the nuclear envelope, respectively. Scale bar equals 5 μm. (**B**) Percentages of telomeres localized at the nuclear periphery in interphase cells. Cells containing one nucleus and one Sid4-mCherry focus (not dissociated) were selected as mitotic interphase cells. Telomeres were scored as localized at the nuclear periphery when the distance between the brightest Taz1-YFP focus and Bqt3-Cerulean-labeled nuclear membrane was less than 0.4 μm using SoftWoRks (Applied Precision; GE Healthcare, Chalfont St Giles, UK). (**C**) Middle z-section image of the cells in mitotic interphase. Example of telomeres detached from the nuclear envelope (left) and telomeres localized at the nuclear periphery (right). Scale bar equals 5 μm. Additional file 4:
**Reduced phosphorylation of Rap1 in** ***taz1***∆. Separation of phosphorylated Rap1-3xPK on a Phos-tag gel from *taz1*∆ synchronized meiotic cell fractions at indicated time. Anti-Cdc2 (CDK) and anti-Cdc13 (Cyclin B) antibodies were used as a loading control and meiosis synchronicity marker, respectively. Note that Taz1 is required for Rap1 localization at telomeres. Additional file 5:
**Fission yeast strain list.**
Additional file 6:
**Technical triplicates of Phos-tag gel related to Fig.** **1d** **.**
Additional file 7:
**Mass spectrometry and microscopy methods.**
Additional file 8:
**Mass spectrometry data for identified phosphopeptides.**

## Abbreviations

AD: Activation domain
BD: Binding domain
SPB: Spindle pole body
