# Supplementary material for: Telomere protein Rap1 is a charge resistant scaffolding protein in chromosomal bouquet formation
Source: BMC Biol. 2015 Jun 10;13:37. doi: 10.1186/s12915-015-0149-x (PMC4660835; doi:10.1186/s12915-015-0149-x)
Supplement: Additional file 1: — List of Rap1 phosphorylation sites identified in the study. [file 12915_2015_149_MOESM1_ESM.pdf]

Additional file 1.

Phospho-site	Phosphopeptides/ total peptides		Identified in mitotic cells *	Bqt4 binding inhibitory effect *
	3.5hrs	4.5hr		
S 180	0/29	1/41	✓	
S 198	1/17	1/30		
T 206	0/17	1/20		
S 212	4/17	5/20		
S 213	9/17	13/20	✓	✓
S 236	0/45	6/78	✓	
S 329	5/10	1/12		
S 348	0/27	2/56	✓	
S 350	3/27	19/56	✓	
T 354	0/27	3/56	✓	
S 357	-	3/4		
T 358	-	1/4		
S 374	5/37	15/51	✓	
T 378	12/33	14/51	✓	✓
S 380	1/34	2/51	✓	
S 387	1/28	3/33		
S 389	2/28	10/33	✓	
S 408	1/18	1/25		
S 410	1/18	1/25		
S 418	0/18	1/25		

S 422	9/18	14/25	✓	✓
S 453	-	1/3		
S 456	0/11	4/98	✓	✓
S 461	0/11	1/98		
S 489	0/2	1/1		
S 497	1/6	1/11		
S 509	0/24	1/41	✓	
S 510	2/24	3/41		
S 512	0/24	3/41		
S 513	9/24	12/41	✓	✓
S 538	13/29	15/26	✓	
S 549	4/16	6/23	✓	
S 552	0/16	2/23	✓	
T 561	2/26	1/43		
S 562	0/26	1/43	✓	

* Data is taken from Fujita *et al.* [15].
