# Supplementary material for: Telomere protein Rap1 is a charge resistant scaffolding protein in chromosomal bouquet formation
Source: BMC Biol. 2015 Jun 10;13:37. doi: 10.1186/s12915-015-0149-x (PMC4660835; doi:10.1186/s12915-015-0149-x)
Supplement: Additional file 5: — Fission yeast strain list. [file 12915_2015_149_MOESM5_ESM.pdf]

Additional file 5.

KTP strain	Genotype
1	*h⁻*
79	*h^{90}*
341	*h^{90} leu1-32 ura4-D18 his3-D1 lys1-131 rap1::$ura4^{+}$ ccq1-YFP:kanMX6 lig4::LEU2 Sid4-mCherry:natMX6*
1170	*h-/h- ade6-M210/M216 rap1-3xPK/rap1-3xPK:natMX6/natMX6 pat1-114/pat1-114 taz1::hygMX6/taz1::kanMX6 $aur1^{R}$mat-Pc/+*
1473	*h^{-}/h^{-} ade6-M210/M216 rap1-3xPK:natMX6/rap1-3xPK:natMX6 pat1-114/pat1-114 $aur1^{R}$:mat-Pc/+*
1900	*h^{90} ade6-M216 leu1-32 ura4-D18 lys1-131 rap1::$ura4^{+}$*
2712	*h^{90} leu1-32 ura4-D18 his3-D1 taz1-YFP:hygMX6 sid4-mCherry:natMX6*
2849	*h^{90} leu1-32 ura4-D18 his3-D1 lys1-131 hht1-Cerulean:$ura4^{+}$ sid4-mCherry:natMX6 rap1-3xflag-YFP:kanMX6*
2850	*h^{90} leu1-32 ura4-D18 his3-D1 lys1-131 hht1-Cerulean:$ura4^{+}$ sid4-mCherry:natMX6 rap1(DD337AA)-3xflag-YFP:kanMX6*
2843	*h^{90} ade6-M216 leu1-32 ura4-D18 lys1-131 PK-rap1(32E):kanMX6*
2867	*h^{90} ade6-M210 leu1-32 ura4-D18 his3-D1 PK-rap1(32E):kanMX6 taz1-YFP:hygMX6 sid4-mCherry:natMX6*
3050	*h^{90} leu1-32 lys1-32 ura4-D18 rap1::$ura4^{+}$ taz1-YFP:hygMX6 sid4-mCherry:natMX6*
3081	*h90 leu1-32 ura4-D18 his3-D1 taz1-YFP:hygMX6 sid4-mCherry:natMX6 bqt3-Cerulean:ura4+*
3205	*h^{90} ade6-M216 leu1-32 ura4-D18 lys1-131 PK-rap1(32E):kanMX6*
3206	*h^{90} leu1-32 lys1-32 ura4-D18 taz1-YFP:hygMX6 sid4-mCherry:natMX6 PK-rap1(32A):kanMX6*
3277	*h^{90} ade6-M216 leu1-32 ura4-D18 lys1-131 PK-rap1(17E):kanMX6*
3278	*h^{90} leu1-32 lys1-32 ura4-D18 taz1-YFP:hygMX6 sid4-mCherry:natMX6 PK-rap1(17E):kanMX6*
3350	*h^{90} leu1-32 lys1-32 ura4-D18 PK-rap1(17A):kanMX6 taz1-YFP:hygMX6 sid4-mCherry:natMX6*
3351	*h^{90} ade6-M216 leu1-32 ura4-D18 lys1-131 PK-rap1(17A):kanMX6*
3442	*h+ ade6-M210 leu1-32 ura4-D18 his3-D1 PK-rap1(DD337AA):kanMX6*

3447	*h^{90} ade6-M210 leu1-32 ura4-D18 lys1-131 rap1(DD337AA)-3xflag-YFP:kanMX6 taz1-mCherry:natMX6 sid4-Cerulean:hygMX6*
3448	*h^{90} ade6-M210 leu1-32 ura4-D18 his3-D1 lys1-131 rap1-YFP:kanMX6 taz1-mCherry:natMX6 sid4-Cerulean:hygMX6*
3607	*h90 ade6-M210 leu1-32 ura4-D18 his3-D1 taz1-YFP:hygMX6 sid4-mCherry:natMX6 bqt3-Cerulean:ura4+ PK-rap1(DD337AA):kanMX6*
3608	*h- leu1-32 ura4-D18 his3-D1 taz1-YFP:hygMX6 sid4-mCherry:natMX6 bqt3-Cerulean:ura4+ rap1::kanMX6*
3609	*h90 ade6-M210 leu1-32 ura4-D18 his3-D1 taz1-YFP:hygMX6 sid4-mCherry:natMX6 bqt3-Cerulean:ura4+ PK-rap1(32E):kanMX6*
3610	*h90 ura4-D18 his3-D1 taz1-YFP:hygMX6 sid4-mCherry:natMX6 bqt3-Cerulean:ura4+ PK-rap1(32A):kanMX6*
3611	*h- pat1-114 PK-rap1:kanMX6*
3614	*h- pat1-114 PK-rap1-32A:kanMX6*
3615	*h- pat1-114 PK-rap1-32E:kanMX6*
