# Supplementary material for: Telomere protein Rap1 is a charge resistant scaffolding protein in chromosomal bouquet formation
Source: BMC Biol. 2015 Jun 10;13:37. doi: 10.1186/s12915-015-0149-x (PMC4660835; doi:10.1186/s12915-015-0149-x)
Supplement: Additional file 7: — Mass spectrometry and microscopy methods. [file 12915_2015_149_MOESM7_ESM.pdf]

Mass spectrometry methods

In-gel digestion

Immunoprecipitated proteins were run into 1D SDS-PAGE gels and visualised with MS-compatible Coomassie stain (GelCode Blue Safe Protein Stain, Thermo Scientific). Briefly, excised gel bands were diced and reduced with 5 mM TCEP, free cysteines were alkylated with 10 mM iodoacetamide, and the proteins were subjected to digestion with modified porcine Trypsin (Promega, Madison, WI). After digestion, the proteolytic peptides were extracted with acetonitrile and triethylammonium bicarbonate washes, the solution dried *in vacuo* and reconstituted in 0.1% formic acid for LC-MS/MS.

LC-MS/MS

Reversed phase chromatography was performed using an HP1200 platform (Agilent, Wokingham, UK). Forty percent of each sample was analysed as a 6 μL injection, spiked with a known concentration of a three-peptide QC mixture. Peptides were resolved on a 75 μm I.D. 15 cm C18 packed emitter column (3 μm particle size; NIKKYO TECHNOS CO.,LTD, Tokyo, Japan) over 30 min using a linear gradient of 96:4 to 50:50 buffer A:B (buffer A: 2% acetonitrile/0.1% formic acid; buffer B: 80% acetonitrile/0.1% formic acid) at 250 nL/min. Peptides were ionised by electrospray ionisation using 1.8 kV applied immediately pre-column *via* a microtee built into the nanospray source. The sample was infused into an LTQ Velos Orbitrap mass spectrometer (Thermo Fisher Scientific, Hemel Hempstead, UK) directly from the end of the tapered tip silica column (6-8 μm exit bore). The ion transfer tube was heated to 200 ˚C and the S-lens set to 60%. MS/MS were acquired using data dependent acquisition based on a full 30,000 resolution FT-MS scan with preview mode disabled and internal lock mass calibration against the polysiloxane ion 445.120025. The top 10 most intense ions were fragmented by collision-induced dissociation and analysed using enhanced ion trap scans. Precursor ions with unknown or single charge states were excluded from selection. The automatic gain control was set to 1,000,000 for FT-MS and 30,000 for IT-MS/MS, full FT-MS maximum inject time was 500ms and normalised collision energy was set to 35% with an activation time of 10 min. Multistage activation (MSA) was used to target phosphoserine/threonine peptides by fragmenting precursor ions undergoing neutral

loss of 32.70, 49.00, 65.40 and 98.00 m/z, corresponding to neutral loss of phosphate, if observed in the top 3 most intense fragment ions. MS/MS was acquired for selected precursor ions with a single repeat count followed by dynamic exclusion with a 10ppm mass window for 10 sec with a repeat count after 5 sec based on a maximal exclusion list of 500 entries.

Database searching

Raw MS/MS data was submitted for database searching using Proteome Discoverer v1.3 (Thermo Scientific) and Mascot V2.3 (Matrix Science). The following Mascot search parameters were used: databases SwissProt: SwissProt_040511a (526,969 sequences) and Uniprot_cu_Spombe (4,983 sequences); enzyme specificity: Trypsin (KR) 2 missed cleavages; mass tolerance: Precursor: 5 ppm Fragment: 0.25 Da; variable modifications: Acetyl (Protein N-term), Carbamidomethyl (C), Oxidation of Methionine, PyroGlu (Peptide N-term Q), Mono/di (KR) /tri methylation (K), Phosphorylation (ST). MS/MS-based peptide and protein identifications were grouped and validated using Scaffold v3.6 (Proteome Software Inc., Portland, OR). Protein identifications were automatically accepted if they contained at least 2 unique peptides assigned with at least 95% confidence by Peptide Prophet.

Microscopy and live cell imaging

Microscopy was performed as described previously [5]. Live analysis was carried out by adhering cells to 35mm glass culture dishes (MatTek) precoated with 0.2mg/ml soybean lectin (Calbiochem) and immersing them in EMM-N media. Imaging was carried out with a DeltaVision Elite (Applied Precision) comprising an Olympus IX71 inverted fluorescent microscope, and Olympus UPlanSApo 100x, NA1.40, oil immersion objective and a CoolSNAP HQ2 camera. Culture dishes were incubated in an Environmental Chamber at 28 ˚C. For live cell imaging, mCherry, YFP and Cerulean signals from a cell were captured with 1.2 sec (32% filter), 1.5 sec (32% filter) and 0.5 sec (32% filter) exposures using Optical Axis Integration (OAI), which acquires 3.6 μm of z-axis by a continuous z sweep, respectively, and was repeated every 300 sec for approximatery 5 hr. For telomere localisation assay, mCherry, YFP and Cerulean signals from cells were captured with 0.2 sec (32% filter), 0.4 sec (32% filter) and 0.3 sec (32% filter) exposures per plane at a 0.3 μm step size over 11 focal

planes z-sections, respectively. These images were deconvolved and analysed using SoftWoRks (Applied Precision).
